# Fallzahlaufkommen und Qualitätsindikatoren bei der Versorgung des abdominellen Bauchaortenaneurysmas

**DOI:** 10.1007/s00104-020-01303-7

**Published:** 2020-10-23

**Authors:** Y. Carmen Ahmadzadeh, Th. Schmitz-Rixen, D. Böckler, R. T. Grundmann

**Affiliations:** 1 Klinik für Gefäß- und Endovascularchirurgie und des Universitären Wundzentrums, Klinikum der Goethe-Universität, Frankfurt/M, Deutschland; 2 Klinik für Gefäßchirurgie und Endovaskuläre Chirurgie, Universitätsklinikum Heidelberg, Heidelberg, Deutschland; 3 Deutsches Institut für Gefäßmedizinische Gesundheitsforschung (DIGG) der Deutschen Gesellschaft für Gefäßchirurgie und Gefäßmedizin, Berlin, Deutschland

**Keywords:** Bauchaortenaneurysma, Endovaskuläre Versorgung, Offene Versorgung, Krankenhausfallaufkommen, Qualitätsindikatoren, Abdominal aortic aneurysm, Endovascular repair, Open repair, Hospital case volume, Quality indicators

## Abstract

**Hintergrund:**

Der MTL30 (Mortalität, Transfer, Liegezeit) wurde als Surrogatparameter zur Evaluation der Qualität potenziell komplikationsträchtiger viszeralchirurgischer Eingriffe vorgeschlagen.

**Zielsetzung:**

Es wurde überprüft, inwieweit sich der MTL30 zu den Ergebnissen des Bauchaortenaneurysma(AAA)-Registers des Deutschen Instituts für Gefäßmedizinische Gesundheitsforschung (DIGG) der Deutschen Gesellschaft für Gefäßchirurgie und Gefäßmedizin (DGG) und zum Fallaufkommen der Kliniken korrelieren lässt.

**Material und Methoden:**

Insgesamt 14.282 Patienten wurden endovaskulär (EVAR) und 3923 Patienten offen (OAR) elektiv wegen eines AAA versorgt. Bestimmt wurden Fallaufkommen der behandelnden Kliniken, Klinikletalität, Liegezeit und Verlegung in ein anderes Akutkrankenhaus 30 Tage nach dem Indexeingriff.

**Ergebnisse:**

Die Klinikletalität machte bei EVAR 1,3 %, bei OAR 4,9 % aus (*p* = 0,000), der MTL30 5,0 % vs. 14,4 % (*p* = 0,000). Für EVAR ließ sich keine Beziehung zwischen Fallaufkommen und Klinikletalität (Quintile 1: 1,0 %; Quintile 5: 1,3 %) sowie Fallaufkommen und MTL30 (Quintile 1: 5,3 %; Quintile 5: 5,3 %) nachweisen. Auch bei OAR bestand keine signifikante Beziehung zwischen Fallaufkommen und Klinikletalität (Quintile 1: 5,8 %, Quintile 5: 3,5 %; *p* = 0,505) und Fallaufkommen und MTL30 (Quintile 1: 16,4 %, Quintile 5: 12,2 %, *p* = 0,110). Bei einer Klinikletalität von 7,2 (5–10) % betrug der MTL30 bei OAR 17,6 %. Sowohl bei EVAR als auch bei OAR korrelierte die stationäre Aufenthaltsdauer signifikant mit Klinikletalität und MTL30.

**Diskussion:**

Eine eindeutige Beziehung zwischen Krankenhausfallaufkommen und Klinikletalität ließ sich im AAA-Register des DIGG nicht aufzeigen. Das gleiche galt für den MTL30. Ob demnach der MTL30 gegenüber der Erfassung von Klinikletalität und stationärer Liegezeit als Qualitätsparameter einen Zusatznutzen bietet, muss offenbleiben.

## Abstract (en)

**Background:**

The MTL30 (mortality, transfer, length of stay) was proposed as a surrogate parameter for evaluating the quality of large and potentially complication-prone visceral surgical interventions.

**Objective:**

The aim of this study was to find out to what extent the MTL30 can be correlated with the results of the abdominal aortic aneurysm (AAA) registry of the German Institute for Vascular Health Research (DIGG) of the German Society for Vascular Surgery and Vascular Medicine (DGG) and with the case volume of the participating hospitals.

**Material and methods:**

Elective endovascular abdominal aortic aneurysm repair (EVAR) was performed in 14,282 patients and open repair (OAR) in 3923 patients. Case volume of the treating hospitals, hospital mortality, length of stay and transfer to another acute care hospital were determined 30 days after the index intervention.

**Results:**

The hospital mortality was 1.3% for EVAR and 4.9% for OAR (*p* = 0.000), the MTL30 was 5.0% and 14.4%, respectively (*p* = 0.000). For EVAR, no relationship between case volume and hospital mortality (quintile 1: 1.0%; quintile 5: 1.3%) as well as case volume and MTL30 (quintile 1: 5.3%; quintile 5: 5.3%) could be demonstrated. Also in OAR there was no significant relationship between case volume and hospital mortality (quintile 1: 5.8%, quintile 5: 3.5%; *p* = 0.505) and case volume and MTL30 (quintile 1: 16.4%, quintile 5: 12.2%, *p* = 0.110). With a hospital mortality rate of 7.2% (5–10%) the MTL30 for OAR was 17.6%. In both EVAR and OAR, the length of stay correlated significantly with hospital mortality and MTL30.

**Discussion:**

A clear relationship between hospital case volume and hospital mortality could not be shown in the AAA registry of the DIGG. The same was true for the MTL30. It remains to be seen whether the MTL30 offers an additional benefit compared to the recording of hospital mortality and inpatient length of stay as a quality parameter.

## Hintergrund

Unter der Vorstellung, dass eine komplikationslos anzusehende komplexe viszeralchirurgische Operation unabhängig von der Art der Operation a) vom Patienten überlebt wird, b) durchgehend in einem Krankenhaus durchgeführt wird und c) mit einer Krankenhausverweildauer von unter 30 Tagen einhergeht, haben Wiegering et al. [13] den sog. MTL30 als Surrogatparameter zur Evaluation der Qualität großer und potenziell komplikationsträchtiger chirurgischer Eingriffe vorgeschlagen. Der MTL30 gilt als eingetreten, wenn ein Patient am 30. Tage nach dem Indexeingriff a) verstorben ist, b) noch in stationärer Behandlung ist oder c) in ein anderes Akutkrankenhaus verlegt wurde. Der Marker spiegelt somit zum einen die Ergebnisqualität mit Letalität und Schwere der Komplikationen als auch partiell die Strukturqualität wie ein dauerhaftes Vorhandensein eines fachärztlichen viszeralchirurgischen Hintergrundes (24 h/365 Tage) oder die Möglichkeit einer Computertomographie wider (z. B. in der Chirurgie des Kolon- oder Rektumkarzinoms [6, 14] oder Chirurgie des Pankreas [12]). Für andere als viszeralchirurgische Eingriffe ist der MTL30 bisher als kombinierter Qualitätsparameter nicht propagiert worden. Die vorliegende Untersuchung stellte sich deshalb die Frage, ob der MTL30 auch als Qualitätssurrogatparameter im Bauchaortenaneurysma(AAA)-Register des Deutschen Instituts für Gefäßmedizinische Gesundheitsforschung (DIGG) der Deutschen Gesellschaft für Gefäßchirurgie und Gefäßmedizin (DGG) zur Anwendung kommen könnte. Dies zum einen unter der Vorstellung, dass Klinikletalität und Komplikationsraten – zumindest bei offener Versorgung (OAR) des AAA – nicht unbeträchtlich sind und dass zum anderen die Ergebnisse auch von der Klinikstruktur bestimmt werden. Zwar können keine definitiven Mindestmengen gefordert werden, jedoch besteht eine signifikante inverse Beziehung zwischen Krankenhausfallaufkommen und Klinikletalität bei Versorgung des intakten AAA (iAAA), die allerdings bei OAR sehr viel ausgeprägter als bei endovaskulärer Versorgung (EVAR) des Aneurysmas ist (Übersicht in [5]). Wir haben deshalb in der vorliegenden Analyse die Registerergebnisse der letzten Jahre hinsichtlich des MTL30 überprüft und dabei untersucht, ob sich dieser Parameter zum Fallaufkommen einer Klinik korrelieren lässt.

## Material und Methodik

Das multizentrische Patientenkollektiv dieser retrospektiven Datenauswertung entstammt dem AAA-Register des DIGG der DGG der Jahre 2013 bis 2017. Alle teilnehmenden Kliniken haben in diesem Register ihre Patienten auf freiwilliger Basis prospektiv dokumentiert. Die Angaben zu Patienten und ihren Klinikaufenthalten wurden pseudonymisiert in das AAA-Register eingetragen. Betrachtet wurden in der vorliegenden Arbeit alle Patienten mit elektiver Versorgung eines nichtrupturierten, symptomatischen oder asymptomatischen Bauchaortenaneurysmas, bei denen die Angaben zu Aneurysmadurchmesser, Klinikletalität, Liegedauer und Transfer in ein anderes Krankenhaus vorhanden waren. Weiterhin wurden nur Kliniken bei der Analyse berücksichtigt, die mindestens zwei EVAR bzw. zwei OAR jährlich dokumentiert haben. Klinische Angaben zu dem Patientenkollektiv finden sich in Tab. 1. Wie ersichtlich, wurden wesentlich mehr Patienten mit EVAR (*n* = 14.282) als mit OAR (*n* = 3923) versorgt und es führten auch weniger Kliniken OAR (*n* = 158) als EVAR (*n* = 212) durch. Allerdings beteiligten sich an dem Register bei EVAR nur 31 Kliniken über die gesamte Dauer von 5 Jahren, bei OAR waren es 16 Kliniken.

**Table 1:** 

Parameter	EVAR	OAR	*P*
Patienten, *n*	14.282	3923	–
Teilnehmende Kliniken, *n*	212	158	–
Männer, *n* (%)	12.382 (86,7)	3275 (83,5)	0,000
Frauen, *n* (%)	1900 (13,3)	648 (16,5)	0,000
Patienten ≥80 Jahre, *n* (%)	3194 (22,4)	360 (9,2)	0,000
Patienten <80 Jahre, *n* (%)	11.088 (77,6)	3562 (90,8)	0,000
Alter insgesamt, in Jahren MW ± SD, Median	73,00 ± 8,43, 74	69,08 ± 8,54, 70	0,000
Alter der Männer, in Jahren MW ± SD, Median	72,67 ± 8,40, 74	70,49 ± 8,79, 70	0,000
Alter der Frauen, in Jahren MW ± SD, Median	75,19 ± 8,35, 76	68,80 ± 9,48, 72	0,000
AAA-Durchmesser Männer, in mm MW ± SD, Median	54,95 ± 11,22, 54	57,26 ± 13,41, 55	0,000
AAA-Durchmesser Frauen, in mm MW ± SD, Median	51,59 ± 11,70, 52	54,39 ± 12,29, 53	0,000
Durchschnittliche Liegedauer, in Tagen MW ± SD	9,89 ± 15,63	17,49 ± 24,30	0,000
ASA ≥3, *n* (%)	10.396 (72,8 %)	2854 (72,8 %)	0,960
Z. n. Myokardinfarkt, *n* (%)	2372 (16,6 %)	637 (16,2 %)	0,580
Renale Begleiterkrankung, *n* (%) ^a^	3096 (21,7 %)	916 (23,3 %)	0,025
Z. n. Apoplex, *n* (%)	1382 (9,7 %)	355 (9,0 %)	0,236

*EVAR* endovaskuläre Aneurysmaversorgung, *OAR* offene Aneurysmaversorgung

^a^Unter „renale Begleiterkrankung“ wurden alle Patienten erfasst mit Z. n. Nieren Tx, Z. n. Nephrektomie und solche, die eine eingeschränkte Nierenfunktion mit einer GFR <90 ml/min (ab Stadium 2) aufwiesen

Es wurde für EVAR und OAR separat überprüft, inwieweit die perioperativen Ergebnisse vom jährlichen Fallaufkommen der teilnehmenden Kliniken abhängig waren. Hierzu wurden die Kliniken bei EVAR von Nr. 1 bis 212 und bei OAR von Nr. 1 bis 158 entsprechend dem ansteigenden jährlichen Fallaufkommen gereiht und dann in Quintile unterteilt, mit Klinikquintile 1 mit dem geringsten und Klinikquintile 5 mit dem höchsten Fallaufkommen. Die Mittelwerte der Querschnittsdaten einer Klinik wurden individuell erstellt, in dem für jede Klinik ihr jährliches Fallaufkommen berechnet wurde. Eine Klinik kann damit nur *einem* Klinikquintil zugehören.

### Statistik

Die Analyse erfolgte primär mit dem Tabellenkalkulationsprogramm Excel für Windows (Microsoft Excel 2019 für Office 365, Version 1908, One Microsoft Way, Redmond, WA 98052-6399, USA). Das Statistikprogramm IBM (Armonk, NY, USA) SPSS Statistics für Windows (Statistical Package for Social Sciences, Version 24) wurde zusätzlich für spezifische Fragestellungen verwendet. Die Gruppen wurden mittels χ^2^-Test auf signifikante Unterschiede überprüft, wobei *p* < 0,05 als Signifikanzniveau gewählt wurde. Die Korrelationen zwischen den einzelnen Parametern wurde mittels Pearson-Korrelationskoeffizienten (r) berechnet. Hierbei wurden Werte <0,3 als schwacher Zusammenhang, Werte zwischen 0,3 und 0,6 als mäßiger Zusammenhang und Werte >0,6 als starker Zusammenhang interpretiert, eine Signifikanz von *p* < 0,05 vorausgesetzt.

## Ergebnisse

### Gesamtkrankengut

Klinikletalität, Fälle mit MTL30, Fälle mit Liegedauer ≥30 Tage und Fälle mit Transfer in ein anderes Krankenhaus sind vergleichend für EVAR und OAR in Tab. 2 wiedergegeben. Die Ergebnisse waren für alle Parameter nach EVAR signifikant besser als nach OAR, mit einer Klinikletalität von 1,3 % vs. 4,9 % und einem MTL30 von 5,0 % vs. 14,4 %.

**Table 2:** 

Parameter	EVAR (*n* = 14.282)	OAR (*n* = 3923)	*p*
MTL30-Fälle, *n* (%)	719 (5,0)	566 (14,4)	0,000
Sterbefälle, *n* (%)	180 (1,3)	191 (4,9)	0,000
Fälle mit Transfer in eine andere Klinik, *n* (%)	207 (1,4)	102 (2,6)	0,000
Fälle mit Liegedauer über ≥30 Tage, *n* (%)	384 (2,7)	328 (8,4)	0,000

*EVAR* endovaskuläre Aneurysmaversorgung, *OAR* offene Aneurysmaversorgung

### Abhängigkeit der Ergebnisse vom Fallaufkommen

Für EVAR ließ sich keine Beziehung zwischen Fallaufkommen und Ergebnis nachweisen, das galt für Klinikletalität (Quintile 1: 1,0 %; Quintile 5: 1,3 %) und MTL30 (Quintile 1: 5,3 %; Quintile 5: 5,3 %) gleichermaßen (Tab. 3). Auch bei OAR bestand keine signifikante Beziehung zwischen Fallaufkommen und Outcome: Klinikletalität Quintile 1: 5,8 %, Quintile 5: 3,5 %; *p* = 0,505; MTL30 Quintile 1: 16,4 %, Quintile 5: 12,2 %, *p* = 0,110 (Tab. 4).

**Table 3:** 

Klinikquintile	Anzahl Kliniken	Fallzahl/Jahr, *n* (Median)	Sterbefälle, *n* (%)	Fälle MTL30, *n* (%)	Fälle Liegedauer ≥30 Tage, *n* (%)	Fälle Transfer, *n* (%)
Q 1, *n* = 1071	44	2–11,5 (8)	11 (1,0)	57 (5,3)	42 (3,9)	9 (0,8)
Q 2, *n* = 2012	43	11,8–16,0 (13,8)	21 (1,0)	98 (4,9)	58 (2,9)	25 (1,2)
Q 3, *n* = 2609	43	16,3–22,8 (19,2)	33 (1,3)	127 (4,9)	66 (2,5)	38 (1,5)
Q 4, *n* = 3349	42	23–32,0 (26,1)	46 (1,4)	159 (4,8)	85 (2,5)	35 (1,0)
Q 5, *n* = 5241	40	32,5–114 (43)	69 (1,3)	278 (5,3)	133 (2,5)	100 (1,9)

*EVAR* endovaskuläre Aneurysmaversorgung

**Table 4:** 

Klinikquintile	Anzahl Kliniken	Fallzahl/Jahr, *n* (Median)	Sterbefälle, *n* (%)	Fälle MTL30, *n* (%)	Fälle Liegedauer ≥30 Tage, *n* (%)	Fälle Transfer, *n* (%)
Q 1, *n* = 208	31	2–3 (2,6)	12 (5,8)	34 (16,4)	21 (10,1)	2 (1,0)
Q 2, *n* = 464	31	3,2–5,2 (4,0)	24 (5,2)	78 (16,8)	48 (10,3)	13 (2,8)
Q 3, *n* = 630	30	5,3–6,8 (6,1)	49 (7,8)	104 (16,5)	46 (7,3)	13 (2,0)
Q 4, *n* = 830	33	7–10 (8,5)	44 (5,3)	132 (15,9)	79 (9,5)	20 (2,4)
Q 5, *n* = 1791	33	10,8–53 (17,5)	62 (3,5)	218 (12,2)	134 (7,5)	54 (3,0)

*OAR* offene Aneurysmaversorgung

### Kliniken mit >30 vs. <30 Fälle/Jahr (EVAR + OAR zusammengefasst)

Neunundsiebzig Kliniken (36,6 %) mit insgesamt 10.297 Patienten gaben an, pro Jahr ≥30 Fälle (OAR und EVAR zusammengefasst) zu behandeln (im Mittel 47,9 Fälle/Jahr). Ihnen stehen 137 (63,4 %) Kliniken mit insgesamt 7908 Patienten gegenüber, die pro Jahr weniger als 30 Fälle (OAR und EVAR zusammengefasst) behandelten (im Mittel 17,5 Fälle/Jahr). Die Ergebnisse (≥30 vs. <30 Fälle/Jahr) waren nicht signifikant unterschiedlich: Klinikletalität 1,9 % vs. 2,2 %; MTL30 6,9 % vs. 7,3 %; Liegezeit über 30 Tage 3,7 % vs. 4,2 %; Fälle mit Transfer 2,0 % vs. 1,3 %.

### Kliniken mit <20 vs. >20 Fälle/Jahr (EVAR + OAR zusammengefasst)

Einhundertdreiunddreißig Kliniken (61,6 %) mit insgesamt 14.735 Patienten gaben an, pro Jahr ≥20 Fälle (OAR und EVAR zusammengefasst) zu behandeln (im Mittel 39,2 Fälle/Jahr). Ihnen stehen 83 (38,4 %) Kliniken mit insgesamt 3470 Patienten gegenüber, die pro Jahr weniger als 20 Fälle (OAR und EVAR zusammengefasst) behandelten (im Mittel 13,5 Fälle/Jahr). Die Ergebnisse (≥20 vs. <20 Fälle/Jahr) waren nicht signifikant unterschiedlich: Klinikletalität 2,0 % vs. 2,0 %; MTL30 7,0 % vs. 7,2 %; Liegezeit über 30 Tage 3,8 % vs. 4,3 %; Fälle mit Transfer 1,8 % vs. 1,3 %.

### Kliniken mit Klinikletalität 5–10 % bei OAR

Dreiunddreißig von 158 Kliniken (20,9 %) mit insgesamt 330 Fällen/Jahr (medianes jährliches Fallvolumen pro Klinik *n* = 7) wiesen bei OAR eine Klinikletalität von 5,0–10,0 % auf. Die Klinikletalität betrug in diesem Kollektiv über alle 7,2 %, der MTL30 17,6 %, Fälle mit Liegezeit ≥30 Tage 10,2 % und Transfer ebenfalls 10,2 %.

### Liegezeit und Klinikletalität

#### EVAR

**Figure 1:**
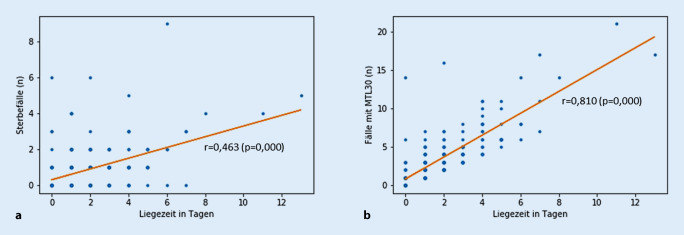

Es gab eine signifikante Beziehung zwischen Dauer der Liegezeit und Klinikletalität sowie MTL30. Dieser Zusammenhang war für den MTL30 ausgeprägter (r = 0,81) als für die Sterblichkeit (r = 0,463; Abb. 1). Des Weiteren wurde die stationäre Aufenthaltsdauer in Quintile unterteilt, nach Kliniken (*n* = 43) mit der längsten (Quintile 1) und Kliniken (*n* = 42) mit der kürzesten Liegezeit (Quintile 5). In Quintile 1 (2013 Patienten) betrug die Liegezeit im Mittel 15,1 ± 35,6 (Median 9) Tage, die Klinikletalität war 1,7 %, der MTL30 7,4 %. Die Vergleichszahlen in Quintile 5 (2845 Patienten) waren: Liegezeit im Mittel 7,2 ± 4,8 (Median 6) Tage, Klinikletalität 1,0 %, MTL30 2,8 %. In Quintile 5 waren damit Klinikletalität (*p* = 0,042) und MTL30 (*p* = 0,000) signifikant günstiger als in Quintile 1.

#### OAR

**Figure 2:**
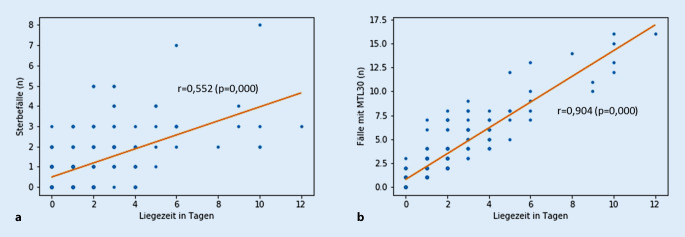

Auch für OAR fand sich eine signifikante Beziehung zwischen Dauer der Liegezeit und Klinikletalität sowie MTL30, wiederum für den MTL30 ausgeprägter (r = 0,904) als für die Sterblichkeit (r = 0,552; Abb. 2). Die stationäre Aufenthaltsdauer, in Quintile unterteilt, ergab 32 Kliniken mit der längsten (Quintile 1) und ebenfalls 32 Kliniken mit der kürzesten Liegezeit (Quintile 5). In Quintile 1 (487 Patienten) betrug die Liegezeit im Mittel 30,1 ± 60,5 (Median 14) Tage, die Klinikletalität war 6,8 %, der MTL30 26,3 %. Die Vergleichszahlen in Quintile 5 (693 Patienten) waren: Liegezeit im Mittel 12,2 ± 5,8 (Median 11) Tage, Klinikletalität 2,2 %, MTL30 5,2 %. In Quintile 5 waren damit Klinikletalität (*p* = 0,000) und MTL30 (*p* = 0,000) signifikant günstiger als in Quintile 1.

## Diskussion

In der vorliegenden Analyse machte die Klinikletalität bei elektiver endovaskulärer Versorgung des AAA 1,3 %, bei offener Versorgung 4,9 % aus. Die signifikant niedrigere Klinikletalität bei EVAR im Vergleich zu OAR war zu erwarten [1] – entsprechend auch dem Registerbericht für das Jahr 2018 [9] – und soll im Zusammenhang mit der Zielsetzung der Arbeit nicht weiter diskutiert werden. So nannten Bulder et al. [2] auf Basis von 51 Studien (189.022 Patienten) nach EVAR eine gepoolte 30-Tage-Letalität von 1,16 % (95 %-Konfidenzintervall 0,92–1,39), verglichen mit 3,27 % (2,71–3,83) nach OAR (*p* < 0,001). Parallel zur Klinikletalität ergaben sich auch signifikante Unterschiede im MTL30, der bei EVAR 5,0 %, bei OAR 14,4 % ausmachte. Da Vergleichsdaten für ein gefäßchirurgisches Krankengut nicht zur Verfügung stehen, muss zur Bewertung des MTL30 auf die viszeralchirurgischen Analysen zurückgegriffen werden. Wiegering et al. [14] nannten für die Chirurgie des Rektumkarzinoms (StuDoQ | Rektumkarzinompopulation) bei 7142 Patienten einen MTL30 von 10,7 %, womit hier die offene Chirurgie des AAA mit einem MTL30 von 14,4 % komplikationsträchtiger als die Chirurgie des Rektumkarzinoms wäre. Inwieweit ein alleiniger Vergleich der Klinikletalitäten zu einer ähnlichen Aussage gekommen wäre, muss offen bleiben, die Klinikletalität wurde von Wiegering et al. für das genannte Krankengut nicht aufgeführt. Die Autoren schlugen aber vor, bei Patienten mit radikaler Primärtumorresektion des Rektums einen MTL30-Wert <20 % zu fordern. Weitere Angaben finden sich für die elektive Pankreatoduodenektomie, Wellner et al. [12] nannten als Qualitätsziele eine Krankenhaussterblichkeit von ≤7 % und einen MTL30-Wert von ≤25 %.

Die Frage ist, welcher MTL30-Grenzwert als Qualitätsziel bei der Versorgung des AAA in unserem Register angegeben werden sollte. Hierzu wurden die Klinken zum einen einem Benchmarking hinsichtlich des Fallaufkommens unterzogen und zum zweiten Qualitätsziele bzw. Fallvorgaben überprüft, die in den Leitlinien der European Society for Vascular Surgery (ESVS; [11]) und der Society for Vascular Surgery (SVS; [3]) für die elektive Versorgung des AAA gefordert werden.

Wie Tab. 3 demonstriert, konnte für EVAR keine Beziehung zwischen Fallaufkommen einer Klinik und Klinikletalität oder MTL30 hergestellt werden, die Klinikquintile 1 mit im Median 8 EVAR/Jahr unterschied sich nicht von Klinikquintile 5 mit im Median 43 EVAR jährlich. Bei der niedrigen Klinikletalität und dem geringen MTL30-Wert (verglichen mit den Angaben zu den oben genannten komplexen viszeralchirurgischen Eingriffen) ist das Ergebnis nicht überraschend und entspricht den Literaturangaben. So kamen Dua et al. [4] zu dem Schluss, dass bei Versorgung des AAA mit EVAR für eine Klinik lediglich 8 EVAR jährlich zu fordern seien, Zettervall et al. [16] schlugen ≥9 EVAR jährlich als Mindestmenge vor. Nimptsch und Mansky [8] werteten deutsche DRG-Daten der Jahre 2009 bis 2014 aus. In dieser Untersuchung (41.678 Patienten mit EVAR) ließ sich zwischen Krankenhausfallaufkommen und Kliniksterblichkeit für EVAR ebenfalls keine signifikante Korrelation erstellen. Die Konsequenz ist, dass sich zum Klinikbenchmarking bei EVAR Angaben zu Fallzahlen und MTL30 nur eingeschränkt eignen. Eher reicht es aus, neben der Klinikletalität in einem Register die Krankenhausaufenthaltsdauer zu erfassen, wobei allerdings die in den US-amerikanischen Registern angegebenen Liegezeiten auf Deutschland nicht direkt übertragbar sind. In einer Erhebung der Vascular Quality Initiative (VQI) der SVS bezeichneten Zettervall et al. [17] bereits eine Aufenthaltsdauer von mehr als 2 Tagen bei 14.510 Patienten mit EVAR als „prolongiert“, was in Abhängigkeit von der Komplikationsrate regional unterschiedlich in 16–43 % der Fall war. Sie sahen in der Bestimmung der Liegezeit einen Qualitätsparameter, da die Aufenthaltsdauer signifikant mit der Komplikationsrate korrelierte. Yin et al. [15] haben Daten der National Surgical Quality Improvement Program(NSQIP)-Datenbank der Jahre 2006 bis 2010 (11.539 Patienten mit EVAR) mit denen der Jahre 2011 bis 2015 (18.537 Patienten mit EVAR) miteinander verglichen. Sie berichteten für beide Beobachtungszeiträume eine gleichbleibend geringe 30-Tage-Letalität von 1,2 %. Bei abnehmender Majorkomplikationsrate von 5,1 % vs. 4,1 % sank parallel die Krankenhausaufenthaltsdauer von 3,2 ± 5,3 Tage auf 2,8 ± 4,3 Tage im nachfolgenden Zeitraum (*p* < 0,001). Wir haben in der vorliegenden Untersuchung deshalb ebenfalls die Aufenthaltsdauer untersucht. Sie korrelierte signifikant mit Klinikletalität und MTL30 (Abb. 1). In der Quintile mit der längsten Liegezeit waren Klinikletalität mit 1,7 % vs. 1,0 % und MTL30 mit 7,4 % vs. 2,8 % signifikant höher als in der Quintile mit der geringsten Liegezeit.

Da mittlerweile bei elektiver Versorgung des AAA generell aufgrund der niedrigeren Klinikletalität – wenn anatomisch/morphologisch möglich – EVAR der Vorzug vor OAR gegeben wird, wie auch hier in 78,5 % der Fälle, ist die ESVS bei Formulierung ihrer Leitlinie einen anderen Weg gegangen, um Mindestmengen zu deklarieren [11]. Sie hat Mindestmengen für den kombinierten Einsatz von OAR plus EVAR definiert. Danach sollte die Versorgung eines AAA nur in Kliniken in Betracht gezogen werden, die wenigstens 30 Fälle jährlich versorgen, unabhängig davon, ob dies EVAR oder OAR ist, und sie sollte *nicht* in Kliniken mit weniger als 20 Fällen jährlich durchgeführt werden (OAR und EVAR zusammengefasst). Auf Registerauswertungen konnte sich die ESVS bei diesen Formulierungen nicht berufen, es handelte sich um Evidenzlevel-B- und -C-Aussagen. Wir haben hier erstmals in einem Register die Ansprüche der ESVS überprüft. Es zeigte sich, dass Kliniken, die EVAR und OAR zusammengefasst mehr als 30-mal pro Jahr durchführten, sich weder in Klinikletalität noch MTL30 von Kliniken unterschieden, die diesen Grenzwert unterschritten. Gleiches galt für die Überprüfung der „Soll-nicht“-Empfehlung der ESVS: Kliniken, die pro Jahr weniger als 20 Fälle (OAR und EVAR zusammengefasst) behandelten, unterschieden sich nicht in Klinikletalität und MTL30 von Kliniken mit höherem Fallaufkommen. Diese Ergebnisse resultieren aus der Tatsache, dass die Mehrzahl der AAA in unserem Register mit EVAR versorgt wurde und die Klinikletalität bei EVAR damit im Wesentlichen die Gesamtletalität des Krankenguts bestimmte.

Auch bei OAR konnten wir im vorliegenden Kollektiv keine signifikante Beziehung zwischen Klinikletalität und MTL30 einerseits und Fallaufkommen andererseits demonstrieren (Tab. 4). Dieses Ergebnis steht im Widerspruch zu anderen Untersuchungen [5] und beruht letztlich auf den geringen Fallzahlen, die uns gemeldet wurden. So kam selbst die höchste Klinikvolumenquintile auf nicht mehr als im Median 17,5 Fälle/Jahr.

Da wir auf Basis der Fallzahlen für den MTL30 bei OAR keinen eindeutigen Grenzwert definieren konnten, der als Qualitätsziel nicht unterschritten werden sollte, haben wir zusätzlich die Forderung der SVS überprüft, der zufolge eine offene Versorgung eines AAA nur in Krankenhäusern mit einer Klinikletalität ≤5 % durchgeführt werden sollte [3]. Wir fanden 30 Kliniken mit einer Klinikletalität von im Mittel 7,2 % (5,0–10,0 %), die das Qualitätsziel der SVS nicht erreichten. Der MTL30 machte in dieser Kohorte 17,6 % aus, sodass wir zunächst einmal, solange keine weiteren Untersuchungen vorliegen, lediglich einen MTL30 von unter <17 % als Qualitätsziel für unser Register bei OAR des AAA nennen können, ein Wert, den allerdings im Durchschnitt sämtliche Volumenquintile erreichten.

Einschränkend muss angemerkt werden, dass große Zentren in dieser Erhebung unterrepräsentiert waren, mit nur 12 von 37 Universitätskliniken. Durchschnittlich wurden jährlich von ca. 130 Krankenhäusern Daten erfasst, obwohl in Deutschland in ca. 500 Krankenhäusern AAA versorgt werden [10]. Ein Selektionsbias ist bei den Registerangaben demnach nicht auszuschließen, die Daten geben nur einen Ausschnitt aus der Versorgungsrealität wieder. Eine externe Validierung der Daten und ein Datenmonitoring waren technisch nicht möglich. Ob, wie aufgefordert, sämtliche Patienten einer Klinik gemeldet wurden, konnte nicht überprüft werden. Wenn demnach in dieser Arbeit vom Fallvolumen gesprochen wird, ist immer das Dokumentationsvolumen gemeint. Auch fragt es sich, ob der MTL30 bei den geringen Unterschieden in den Klinikquintilen als Qualitätsziel tatsächlich der geeignetste Parameter ist (Tab. 3 und 4). In zukünftigen Untersuchungen unseres Registers sollte deshalb vielleicht dem Vorschlag von Hardt et al. [7] gefolgt und der MTL22 als Qualitätsparameter untersucht werden, der Mortalität, Transfer und Liegezeit bereits nach 22 Tagen erfasst. Dieser Parameter war in der Analyse der kolorektalen Karzinomchirurgie dem MTL30 hinsichtlich der Sensitivität bei der Erfassung der postoperativen Morbidität mit 65 % vs. 40 % eindeutig überlegen. Unabhängig davon liegen aber bisher generell zu wenige Registererhebungen vor, die den MTL30 bewertet haben. Dieser Beitrag versteht sich als Ansatz, diese Lücke zu schließen.

## Schlussfolgerung

Eine eindeutige Beziehung zwischen Krankenhausfallaufkommen und Klinikletalität ließ sich im AAA-Register des DIGG nicht aufzeigen. Das gleiche galt für den MTL30, der sich nicht zu dem Fallaufkommen einer Klinik korrelieren ließ. Ob demnach der MTL30 gegenüber der Erfassung von Klinikletalität und stationärer Liegezeit als Qualitätsparameter einen Zusatznutzen bietet, muss offenbleiben.
